# Inactivation of DNA-Binding Response Regulator Sak189 Abrogates β-Antigen Expression and Affects Virulence of *Streptococcus agalactiae*

**DOI:** 10.1371/journal.pone.0010212

**Published:** 2010-04-19

**Authors:** Anastasia S. Rozhdestvenskaya, Artem A. Totolian, Alexander V. Dmitriev

**Affiliations:** Department of Molecular Microbiology, Institute of Experimental Medicine, Saint-Petersburg, Russia; St. Petersburg Pasteur Institute, Russian Federation

## Abstract

**Background:**

*Streptococcus agalactiae* is able to colonize numerous tissues employing different mechanisms of gene regulation, particularly via two-component regulatory systems. These systems sense the environmental stimuli and regulate expression of the genes including virulence genes. Recently, the novel two-component regulatory system Sak188/Sak189 was identified. In *S. agalactiae* genome, it was adjacent to the *bac* gene encoding for β-antigen, an important virulence factor.

**Methodology/Principal Findings:**

In this study, the *sak188* and *sak189* genes were inactivated, and the functional role of Sak188/Sak189 two-component system in regulation of the β-antigen expression was investigated. It was demonstrated that both transcription of *bac* gene and expression of encoded β-antigen were controlled by Sak189 response regulator, but not Sak188 histidine kinase. It was also found that the regulation occurred at transcriptional level. Finally, insertional inactivation of *sak189* gene, but not *sak188* gene, significantly affected virulent properties of *S. agalactiae*.

**Conclusions/Significance:**

Sak189 response regulator is necessary for activation of *bac* gene transcription. It also controls the virulent properties of *S. agalactiae.* Given that the primary functional role of Sak188/Sak189 two-component systems is a control of *bac* gene transcription, this system can be annotated as BgrR/S (*bac* gene regulatory system).

## Introduction

*Streptococcus agalactiae* (group B streptococcus) is an important cause of neonatal invasive infections and variety of diseases in human and animals [1], [2]. This bacterium is able to sense the changing environmental conditions and colonize numerous tissues employing different mechanisms of gene regulation. One mechanism for adaptation to changing environment is transcriptional regulation by two-component regulatory systems (TCSs), a family of systems that are widely distributed among many bacterial genera [3]. These regulatory systems comprise two proteins, sensor histidine kinase, HK, and DNA-binding response regulator, RR. HK is a sensor protein, responding to the environmental changes by autophosphorylating a conserved histidine residue. Subsequently, phosphoryl group is transferred to RR protein resulting in conformational changes in RR molecule. Conformationally modified RR molecule functions as transcriptional regulator (activator or repressor) by binding with promoter regions of the genes through helix-turn-helix (HTH) motif. In many cases, two-component systems play an important role in the regulation of multiple genes encoding products which are essential for adaptation of bacteria to a particular environment and development of bacterial diseases [3]–[5].

In contrast to other pathogenic gram-positive cocci such as *S. pneumoniae* and *S. pyogenes*, *S. agalactiae* has larger number of TCSs. For example, 20 TCSs have been identified after the complete genome sequencing in the strain NEM316 [6], and 17 TCSs–in the strain 2603 V/R [7]. Three of them, CsrRS/CovRS, DltR/DltS, and RgfA/RgfC, have been previously investigated [8]–[12].

*S. agalactiae* CsrRS/CovRS demonstrated high similarity with CsrRS/CovRS of *S. pyogenes* [8]. Inactivation of *covR* in *S. agalactiae* increased transcription levels of hemolysin *cylE* gene and C5a peptidase *scpB* gene, and reduced the level of CAMP factor *cfb* gene [8], [9]. Inactivation of CovRS was also observed at phenotypic level: the mutant strains became less virulent compared to the parental wild-type strain [9]. Transcriptome analysis revealed the CovRS core-regulon which consisted of 39 genes including genes of the stress response, the genes important for adhesion of *S. agalactiae* to the host cells and others [10]. DltR/DltS of *S. agalactiae* is important for transcription of *dlt* operon (*dltA, dltB, dltC*, and *dltD* genes), which is necessary for incorporation of D-alanine residues in lipoteichoic acids. The role of DltR/DltS in *S. agalactiae* virulence was shown by the attenuated virulent properties of *dltA* and *dltR* mutant strains *in vivo* [11]. RgfA/RgfC controls adhesion of *S. agalactiae* to epithelial cells. In addition, inactivation of histidine kinase gene *rgfC* resulted in increased transcription of *scpB* gene [12].

Recently, putative two-component regulatory system genes encoding for sensor histidine kinase (*sak188*) and DNA-binding response regulator (*sak189*) were identified in *S. agalactiae* strains 98-D60C and A909 [13], [14]. *sak188* and *sak189* genes were located on putative pathogenicity island [13] adjacent to the *bac* gene encoding for β-antigen. This surface protein has capacity to bind IgA and factor H of complement, and it is supposed to be an important virulence factor [15]. Sak188 and Sak189 proteins revealed 78% and 83% similarity with sensor histidine kinase HK06 and DNA-binding response regulator RR06 of *S. pneumoniae* strains, respectively [13], [16]–[19]. In *S. pneumoniae* genome, the *hk06* and *rr06* genes are adjacent to *cbpA* gene encoding choline binding protein, CbpA, a surface-exposed protective antigen and virulence factor of *S. pneumoniae* [20], [21]. Similarly to the β-antigen, CbpA has capacity to bind factor H of complement. In addition, some regions of CbpA protein has the certain similarity with β-antigen. Inactivation of RR06 in *S. pneumoniae* resulted in the loss of CbpA expression [22]–[24]. HK06/RR06 was also important for the ability of pneumococci to adhere to epithelial cells *in vitro* and to survive and proliferate in an *in vivo* mouse model [22]. Given the certain similarity in the sequences, functions and relative locations of Sak188/Sak189 and HK06/RR06, and β-antigen and CbpA, we suggested that Sak188/Sak189 TCS affected β-antigen expression and virulent properties of *S. agalactiae* in the same manner as HK06/RR06 affected CbpA in *S. pneumoniae*.

The goal of the present study was to investigate the function of *S. agalactiae* Sak188/Sak189 TCS.

## Materials and Methods

### Bacterial strains and growth conditions

A total of 239 strains from collection of the Institute of Experimental Medicine isolated from human (139 strains) and cows (100 strains) were studied. The *S. agalactiae* strain 168/00 (serotype Ib) was used as parental strain for insertional inactivation of *sak188* and *sak189* genes. *Escherichia coli* strains JM109 and DH5α served as hosts for expression vectors and recombinant plasmids. *S. agalactiae* strains were cultured in Todd-Hewitt Broth (THB) (HiMedia Laboratories Pvt. Ltd., India) or on THB agar supplemented with 5% erythrocytes at 37°C. *E. coli* strains were grown in Luria-Bertani (LB) broth (Sigma, USA) or on 1% LB agar. Antibiotics were used in the following concentrations: 2.5 µg/ml of erythromycin for *S. agalactiae*; 200 µg/ml of erythromycin and 100 µg/ml of ampicillin for *E. coli*.

### DNA techniques

The oligonucleotide primers for PCR and sequencing are listed in the Table 1. Routine DNA techniques were used for nucleic acid analysis. Chromosomal DNA was isolated by the phenol-chloroform extraction [25]. Plasmid DNA was isolated and purified using the AxyPrep Plasmid Miniprep Kit and AxyPrep Plasmid Midiprep Kit (Axygen Biosciences, USA) according to the manufacturer's instructions. PCR was carried out with *Taq* polymerase with initial denaturation of 2 min at 94°C followed by 30 cycles of amplification (30 sec at 94°C, 1 min at 52°C, and 1 min at 72°C). PCR products were purified with AxyPrep DNA Gel Extraction Kit (Axygen Biosciences). Sequencing of PCR products was performed by an ABI 3100 automated DNA sequencer using the Big-Dye Terminator Kit (Applied Biosystems, USA).

**Table 1:** Primers used in the study.

Primer	Nucleotide sequence (5′–3′)	Annealing temperature, °C	Gene/Plasmid
hkFor	GGTCATCCACATTGCCATCTATC	54.8	*sak188*
hkRev	GTCAAAACCTTATCCAATGCCTT	53.3	*sak188*
rrFor	GATATGATTAGAGAGGGAGTAGCTGCT	54.3	*sak189*
rrRev	GACCTGGTTCTTATGTTGGATCAA	53.9	*sak189*
274	GTAAAACGACGGCCAGTG	48.6	pGEM-T Easy
275	CAGGAAACAGCTATGACCATG	49.8	pGEM-T Easy
40/1	AGGAGGGACAGCTGGATATTACG	55.4	pVA891-2
40/2	TCCCATTTAGCCGTCATTTCAG	55.6	pVA891-2
L5	TGTAAAGGACGATAGTGTGAAGAC	49.8	*bac*
L6	CATTTGTGATTCCCTTTTGC	49.7	*bac*
0417-60	TTCGTAAATTTAGTGTAGGAGTAGCT	49.9	*bac*
0417-61	CCTTGATCTGTTTGAGCCATA	49.5	*bac*
BAC5	CTACAGTTGACAAAGAGCCT	56.0	*bac*
0407-172	CACAAATGGAGATGCTGACTAG	49.2	*bac*
SAGAF	CGTCGTGGTATTGAATCAGCTGTT	51.0	*cpn60*
SAGAR	GGATATACGGATTCGCAAGTTCAGAG	53.0	*cpn60*

### SDS-PAGE and western-blotting

*S. agalactiae* strains were grown during 2 and 5 hours, corresponding to middle-exponential and post-exponential phase of growth, respectively, and the cells were harvested by centrifugation. Bacterial cell lysates were prepared by extraction with HCl [26], NaOH [27], and 2-mercaptoethanol [28] and by vortexing of the cell suspensions with glass beads of 0.1 mm in diameter. For isolation of secreted proteins, cells were removed by centrifugation, and 1.8 volume of acetone was added. Secreted proteins were precipitated by centrifugation, resuspended in phosphate-buffered saline (PBS) and dialyzed overnight against the PBS. The same volume of buffer containing 2-mercaptoethanol (10%, w/w) was added to all samples, and the samples were boiled for 10 min.

Bacterial cell lysates and secreted proteins were subjected to sodium dodecyl sulfate-polyacrylamide gel electrophoresis (SDS-PAGE) as described by Laemmli [28]. The gels were stained with Coomassee R-250 (Amresco, USA) or electroblotted onto nitrocellulose membrane Trans-Blot Transfer Medium, 0.45 µm, (Bio-Rad Laboratories, USA) as previously described [29] and probed with conjugated human IgA-horseradish peroxidase.

### RNA isolation

An overnight cultures of *S. agalactiae* strains were inoculated into 40 ml of THB in 50-ml tubes (Fisher Scientific, USA) to an *A*_600_ = 0.08. The cultures were grown to the middle-exponential phase corresponding to 2 hrs of growth. Cultures were centrifuged, and the cells were suspended in 5 ml of *RNAprotect* (QIAGEN, USA). RNA was isolated with an RNeasy Mini Kit (QIAGEN), according to the protocol recommended by the manufacturer. The concentration of RNA was assessed with an SmartSpec™3000 (Bio-Rad Laboratories).

### Quantitative reverse transcriptase (RT)-PCR

Oligonucleotide primers (Table 1) were designed with Primer-BLAST (www.ncbi.nlm.nih.gov/tools/primer-blast). Amplification and detection were done with the ABI Prism 7500 Real-Time PCR System (Applied Biosystems) using Power SYBR® Green RNA-to-C_T_™ 1–Step Kit (Applied Biosystems), as recommended by the manufacturer. Briefly, a total of 20 ng of total RNA was used in the reaction. Reverse transcription was done at 48°C for 30 min, and amplification was performed at the following conditions: activation of AmpliTaq® Gold DNA Polymerase at 95°C for 10 min followed by 40 cycles of denaturation at 95°C for 15 sec and annealing/extension at 60°C for 1 min. Each assay was done in duplicates with two independently isolated RNA samples. The quantity of *bac* gene RNA was normalized to the quantity of *cpn60* gene RNA in each sample, and the mean ± standard error of the mean of independently isolated RNA samples was determined.

### *In vivo* infection model

*S. agalactiae* wild-type strain 168/00 and mutant strains were grown in 40 ml of THB overnight, centrifuged, washed twice with sterile PBS, and resuspended in 4 ml of PBS. Ten-week old (14–16 g), male, white outbread mice (Rappolovo Laboratory, Rappolovo, Russia) were used in the study. They were housed according to standard animal laboratory conditions. Each animal was infected by intra-peritoneal injection of 0.5 ml of PBS containing 10^8^ CFUs of the strain. A total of 15 animals were used in each experimental group.

All the mice were monitored twice a day for up to 10 days. Once each individual animal was died, it was dissected, and the spleen was isolated and homogenized in 1 ml of PBS. In order to determine the number of *S. agalactiae* CFUs in the spleen, ten-fold dilutions of suspensions were grown at THB blood agar plates containing erythromycin when appropriate. As control, injection of PBS (0.5 ml) was applied to one additional group of animals. At the end of survival assay, all the remaining mice were sacrificed, and their spleens were analyzed as described above. All the experimental procedures were done according to the principles and guidelines for the care and use of laboratory animals (National Institutes of Health, USA, and Russian Academy of the Medical Sciences, Russia) and were approved by Saint-Petersburg Institute of Experimental Medicine Animal Care Unit Committee, Russia.

### Nucleotide sequence accession numbers

The nucleotide sequences of *sak188* and *sak189* genes of the strain 168/00 have been deposited into the GenBank database under accession number FJ890928.

## Results

### An occurrence of *bac* gene, *sak188*, *sak189* TCS genes and erythromycin resistance among *S. agalactiae* strains

**Figure 1:**
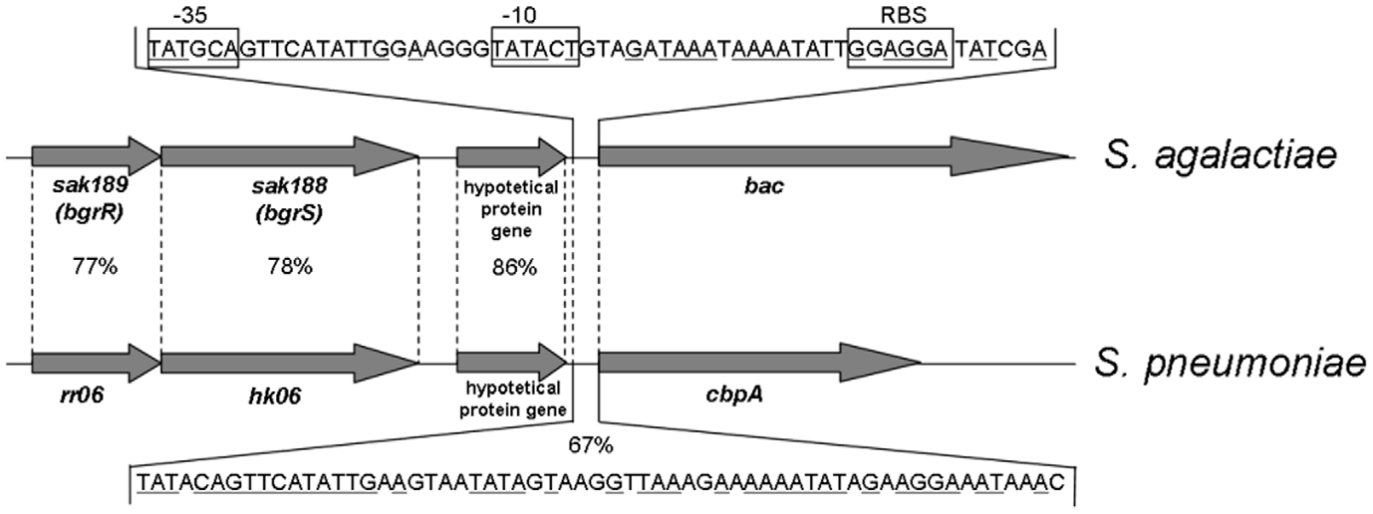
Genomic architecture of analyzed DNA fragments in *S. agalactiae* and *S. pneumoniae*. Promoter regions of the *bac* and *cbpA* genes are shown, and identical nucleotides are underlined. The putative -35 and -10 sequences upstream the *bac* gene and RBS site according to Jerlstrom et al. [44] are indicated.

A total of 239 *S. agalactiae* strains isolated from human and animals were studied. The strains were tested by PCR for the presence of virulence gene *bac* and *sak188* and *sak189* TCS genes, which were previously found to be located on pathogenicity island [13]. As result, 53 strains isolated from pregnant women and 5 strains isolated from the cow's milk were found to be *bac* gene positive. They were selected for further study. PCR analysis of the *bac* gene positive strains revealed that the presence of *bac* gene correlated with the presence of sensor histidine kinase gene *sak188* and DNA-binding response regulator gene *sak189* (data not shown). Genomic architecture of DNA fragments containing *sak188*, *sak189* and *bac* genes in *S. agalactiae* [13], [14], and *rr06*, *hk06* and *cbpA* genes in *S. pneumoniae* [22] is presented in the Fig. 1.

For the purpose of insertional inactivation, the vector pVA891-2 that provides erythromycin (Em) resistance was used. Thus, the strains were additionally screened for sensitivity to Em. As result, most of human *bac* gene positive strains showed resistance or intermediate resistance to Em in the standard diffusion disk test, that is in agreement with previous observations demonstrating an increase in Em resistance in *S. agalactiae* population [30], [31]. One of the strains, 168/00, which demonstrated the large diameter of growth inhibition zone, was selected for further study. In this strain, the *bac* gene has unique allele with the closest similarity with 676a and 676b alleles according to classification of Kong et al. [32].

### Construction and characterization of mutant strains

**Figure 2:**
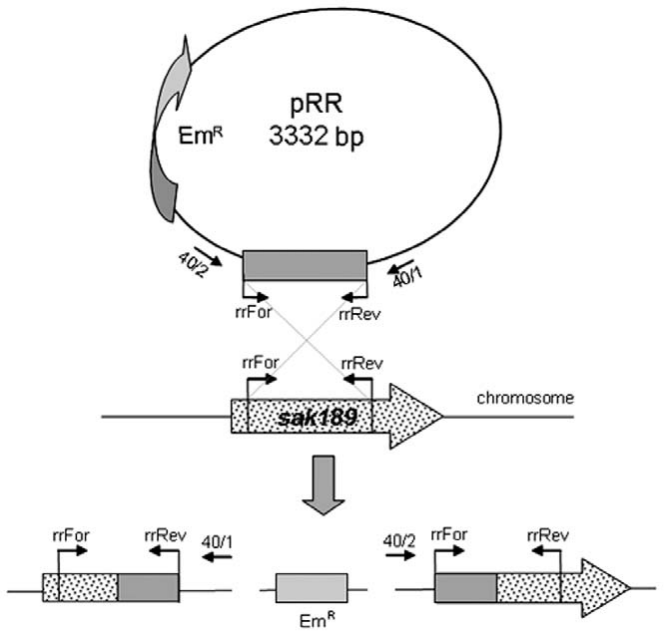
Schematic representation of insertional inactivation of *sak189* gene in *S. agalactiae* strain 168/00. Insertional inactivation of *sak188* gene was done similarly.

To investigate a functional role of the two-component regulatory system Sak188/Sak189 in *S. agalactiae*, the *sak188* and *sak189* genes were inactivated by insertional mutagenesis (Fig. 2). Briefly, DNA fragments of *sak188* and *sak189* genes were PCR amplified using pairs of primers hkFor, hkRev, and rrFor, rrRev (Table 1), respectively. PCR products were cloned into pGEM-T Easy vector (Promega, USA), and the recombinant plasmids named as pGEM-T*sak188* and pGEM-T*sak189* were digested with *Eco*RI. Subsequently, *Eco*RI fragments were cloned into suicide vector pVA891-2, and the recombinant plasmids named as pHK and pRR, respectively, were used to transform *S. agalactiae* strain 168/00 according to the protocol [33]. The recombinant clones were selected on THB agar plates containing 2.5 µg/ml of erythromycin. Construction of the strains was confirmed by PCR and nucleotide sequencing (data not shown). Given that insertional inactivation of RR gene results in inactivation of HK gene [22] which are co-transcribed (Fig. 1), the strain with insertionally inactivated *sak189* gene can be considered as *sak189/sak188* double mutant strain, while the strain with insertionally inactivated *sak188* gene–as *sak188* mutant strain.

Comparative analysis of the wild-type strain 168/00 and mutant strains revealed no difference in morphological properties. The size, shape and color of the colonies, diameter of zone of hemolysis, morphology of the cells and the chain length were similar in all the strains. At the same time the growth curves of mutant strains in THB slightly differed from the growth curve of the strain 168/00 (data not shown). Two time points (2 hrs and 5 hrs) corresponding to the middle-exponential and post-exponential phases of growth, respectively, were selected for further study.

### Effect of insertional inactivation on expression of β-antigen

**Figure 3:**
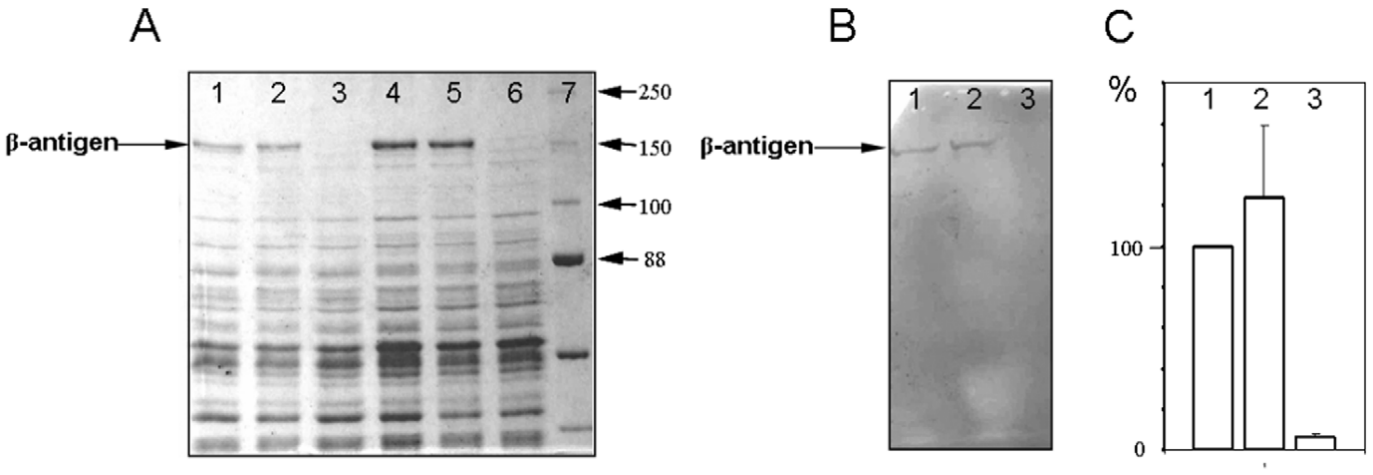
Expression of β-antigen and *bac* gene transcription levels in *S. agalaciae* strains. Expression of β-antigen in the whole cell lysates of the wild-type strain 168/00 and mutant strains after 2 hrs (lanes 1–3) and 5 hrs of growth (lanes 4–6) was analyzed by SDS-PAGE (Panel A) and western-blotting with labeled human IgA (Panel B). Lanes 1 and 4–strain 168/00; lanes 2 and 5–*sak188* mutant strain; lanes 3 and 6–*sak189/sak188* double mutant strain; lane 7–protein ladder (Bio-Rad Laboratories). *bac* gene transcription levels (Panel C) were assessed by quantitative reverse transcriptase (RT)-PCR. Numbers indicate relative differences (%) in transcription levels of *bac* gene in mutant strains compared to transcription level of *bac* gene in wild-type strain. The *bac* gene transcription level was normalized to the *cpn60* gene transcription level in each strain. Mean values and standard errors are shown as results of two independent experiments.

Several approaches were used to prepare the cell lysates of the wild-type strain 168/00 and mutant strains. As result, 10 min boiling of the cells in 2-mercaptoethanol (10%, w/w) was found to be most effective with respect to higher yield and quality of the protein bands observed after SDS-PAGE (data not shown). Subsequently, bacterial cell lysates and secreted proteins after 2 hrs and 5 hrs of growth in THB were analyzed. SDS-PAGE of secreted proteins did not reveal any difference among the wild-type strain and mutant strains (data not shown). However, SDS-PAGE of the cell lysates revealed that at least one protein band of ≈140 kDa was present in the wild-type strain 168/00 and *sak188* mutant strain, and was absent in *sak189/sak188* double mutant strain. Western-blotting with labeled human IgA identified this protein as β-antigen (Fig. 3).

### Effect of insertional inactivation on transcription of *bac* gene

In order to analyze if changes in β-antigen expression are regulated at transcriptional level, the quantitative RT-PCR was performed using RNA samples isolated during the middle-exponential phase corresponding to 2 hrs of growth. As result, transcription of *bac* gene in *sak189/sak188* double mutant strain was 17-fold less than in 168/00 wild-type strain (Fig. 3). On the other hand, the *bac* gene transcription level in *sak188* mutant strain was almost similar to that in the wild-type strain that confirmed the results of SDS-PAGE and western-blotting. Thus, these data demonstrate that Sak189 protein regulates expression of β-antigen at transcriptional level.

### *In vivo* virulent properties of the wild-type strain and mutant strains

**Figure 4:**
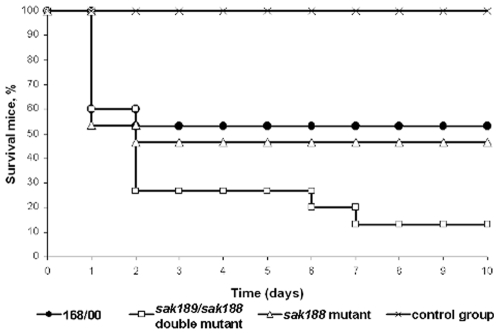
Mortality rates of laboratory mice due to the streptococcal intraperitoneal infection. Mice were infected by intra-peritoneal injection of 0.5 ml of PBS containing 10^8^ CFUs of 168/00 wild-type strain, *sak188* mutant strain and *sak189/sak188* double mutant strain, and analysis of the virulent properties was assessed as described in Materials and Methods. As control, injection of PBS (0.5 ml) was applied to additional group of animals.

Murine intraperitoneal model of streptococcal infection was developed to compare the virulent properties of the strains under study. Mice were injected with 0.5 ml of PBS containing 10^8^ CFUs and observed for 10 days. As seen from the Fig. 4, the mortality rate caused by *sak189/sak188* double mutant strain significantly differed in comparison with that caused by the wild-type and *sak188* mutant strains. Compared to the wild-type strain, the differences in virulent properties were statistically significant in case of *sak189/sak188* double mutant strain (*P*-value <0.02), and not significant in case of *sak188* mutant strain (*P*-value  = 0.73).

## Discussion

Transcriptional regulation of the genes is important process for successful adaptation of pathogenic bacteria to changing environments, particularly, during the interaction of pathogen with human host. In *S. agalactiae*, a total of 21 TCSs are involved in the regulatory network essential for successful colonization, penetration and/or invasion as well as for resistance to human immune system. In this study, we investigated the functional role of Sak188/Sak189 two-component system in regulation of the expression of β-antigen, an important virulence factor of *S. agalactiae*. It was demonstrated that both transcription of *bac* gene and expression of encoded β-antigen were controlled by the Sak189 response regulator, but not Sak188 histidine kinase. It was also found that regulation occurred at transcriptional level. Finally, insertional inactivation of *sak189* gene, but not *sak188* gene, significantly affects virulent properties of *S. agalactiae*.

**Figure 5:**
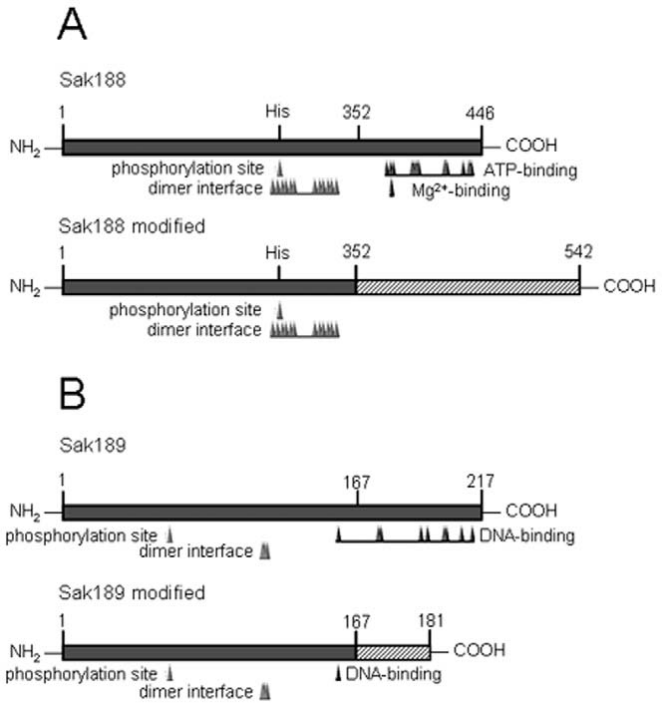
Structures of Sak188 and Sak189 proteins in the wild-type 168/00 strain and mutant strains. The sequence of Sak188 modified protein in *sak188* mutant strain (A) and the sequence of Sak189 modified protein in *sak189/sak188* double mutant strain (B) were deduced, and the functional domains/activities of native proteins and modified proteins were predicted according to BLASTp analysis.

Variety of publications are currently available regarding the occurrence of *bac* gene among *S. agalactiae* strains as well as genetic polymorphism of *bac* gene and use of encoded β-antigen as a vaccine component [32], [34]–[38]. In some strains, the mutations in *bac* gene resulted in expression of truncated non-functional forms of β-antigen [39], [40]. However, the regulatory mechanism of *bac* gene expression has not been studied. In present study, the *sak188* and *sak189* genes of *S. agalactiae* were inactivated. Given that insertional inactivation does not delete the entire gene from the chromosome and often results in truncated or modified protein, we analyzed which functional activities of Sak188 and Sak189 proteins could potentially retain if the modified proteins expressed. Comparative analysis of Sak188 and Sak189 amino acid sequences in the wild-type strain 168/00 and deduced protein sequences in mutant strains predicted the replacement of 95 amino acids at the C-terminal end of Sak188 protein by 191 amino acids, and replacement of 51 amino acids at the C-terminal end of Sak189 protein by 15 amino acids (Fig. 5). Importantly, 8 out of 9 amino acids, which composed HTH motif presumably involved in DNA-binding according to BLASTp analysis (http://blast.ncbi.nlm.nih.gov/Blast.cgi), were lost in Sak189 protein due to insertional mutagenesis. Similarly, ATP-binding and Mg^2+^-binding sites located at C-terminal end of Sak188, which are essential for phosphorylation, were lost in Sak188 protein due to the mutagenesis. Theoretically, in both *sak188* mutant strain and *sak189/sak188* double mutant strain, the Sak188/Sak189 TCS can be inactive.

In *S. pneumoniae*, inactivation of the response regulator RR06 (83% similarity with Sak189), which binds promoter region of *cbpA*, repressed transcription of the *cbpA* gene [22]. In this study, western blotting and quantitative RT-PCR revealed that DNA-binding response regulator Sak189 is necessary for β-antigen expression suggesting direct binding of Sak189 with *bac* gene promoter. This is not surprising given the high similarity in the sequences, functions and relative locations of Sak188/Sak189 and HK06/RR06, and β-antigen and CbpA (Fig. 1).

Importantly, insertional inactivation can have a polar effect on the genes located downstream. In this study, both *sak188* and *bac* genes located downstream the *sak189* gene (Fig. 1). As seen from the Fig. 3, insertional inactivation of *sak189* affected *bac* gene transcription, while insertional inactivation of the *sak188* did not affect. Thus, we can conclude that *sak188* and *bac* gene are not in the operon, and repression of *bac* gene transcription in *sak189/sak188* double mutant strain is not due to the polar effect.

Given that insertional inactivation of *sak188* gene does not affect β-antigen expression, we suggest that β-antigen expression can be activated independently of Sak188, perhaps through small-molecular-weight phosphodonors such as acetyl phosphate or other noncognate histidine kinases. Thus, we can not rule out the possibility of a cross-talk between Sak189 response regulator and sensor kinase(s) in *S. agalactiae*, and Sak189 can potentially recruit another histidine kinase for proper functioning. The cross-talk among different TCSs has been described for many bacterial species [3] including pathogenic streptococci. For example, in *S. pneumoniae* inactivation of histidine kinase HK06 (78% similarity with Sak188), in contrast to RR06 (83% similarity with Sak189), did not repress transcription of the adjacent *cbpA* gene [22] indicating a cross-talk between RR06 and noncognate histidine kinase(s).

Intraperitoneal infection of laboratory mice demonstrated that Sak189 DNA-binding response regulatory protein is important for virulent properties of *S. agalactiae*. It could be predicted given that inactivation of other *S. agalactiae* TCSs (CovS/CovR, DltR/DltS, RgfA/RgfC) resulted in the changes of virulent properties, however, in all these studies the mutant strains became less virulent compared to the wild-type strains [8]–[12]. In the present study, insertional inactivation of *sak189* gene abrogated β-antigen expression but resulted in increased virulence (Fig. 4). Thus, we suggest that β-antigen located on the bacterial surface can interfere with the disease process and play a role of anti-virulent factor (at least, in mice) similarly to hyaluronate lyase of *S. pyogenes* [41]. It is also in agreement with results of Kong et al. who demonstrated that presence of shorter variants of β-antigen is associated with increased virulence [32].

It is also possible that *bac* gene encoding for β-antigen is not only target for Sak188/Sak189 TCS. The difference among the growth curves of the wild-type and mutant strains (data not shown) indicate that in addition to *bac* gene, some metabolic genes can be controlled by Sak188/Sak189 TCS. This suggestion is confirmed by the numerous studies demonstrating the genome-wide location of the genes affected by TCSs [10], [42], [43], and set of the genes controlled by Sak188/Sak189 (TCS regulon) can be identified using microarray technology.

As seen from the data of this study, transcription of *bac* gene and expression of encoded β-antigen are the primary genotypic/phenotypic properties controlled by Sak188/Sak189 TCS. Therefore, it is reasonable to annotate this system as BgrR/S (*bac* gene regulatory system), and DNA-binding response regulator Sak189 and sensor histidine kinase Sak188 as BgrR and BgrS proteins, respectively.

In conclusion, the data presented here characterize the functional role of *S. agalactiae* Sak188/Sak189 two-component system. Both transcription of *bac* gene and expression of encoded β-antigen are controlled by Sak189 response regulator, and the regulation occurs at transcriptional level. Inactivation of histidine kinase Sak188 does not affect transcription of *bac* gene and expression of β-antigen, that suggests the cross-talk between Sak189 and other sensor kinase(s) in *S. agalactiae*. Finally, Sak189, but not Sak188, controls virulent properties of *S. agalactiae in vivo*.

## References

[1] Keefe GP (1997). *Streptococcus agalactiae* mastitis: a review.. Can Vet J.

[2] Facklam RR, Washington JA, Balows A, (1991). Streptococcus and related catalase-negative gram-positive cocci.. Manual of Clinical Microbiology..

[3] Stock AM, Robinson VL, Goudreau PN (2000). Two-component signal transduction.. Annu Rev Biochem.

[4] Lamarche MG, Wanner BL, Crépin S, Harel J (2008). The phosphate regulon and bacterial virulence: a regulatory network connecting phosphate homeostasis and pathogenesis.. FEMS Microbiol Rev.

[5] Kleerebezem M, Quadri LE, Kuipers OP, de Vos WM (1997). Quorum sensing by peptide pheromones and two-component signal-transduction systems in Gram-positive bacteria.. Mol Microbiol.

[6] Glaser P, Rusniok C, Buchrieser C, Chevalier F, Frangeul L (2002). Genome sequence of *Streptococcus agalactiae*, a pathogen causing invasive neonatal disease.. Mol Microbiol.

[7] Tettelin H, Masignani V, Cieslewicz MJ, Eisen JA, Peterson S (2002). Complete genome sequence and comparative genomic analysis of an emerging human pathogen, serotype V *Streptococcus agalactiae*.. Proc Natl Acad Sci U S A.

[8] Lamy MC, Zouine M, Fert J, Vergassola M, Couve E (2004). CovS/CovR of group B streptococcus: a two-component global regulatory system involved in virulence.. Mol Microb.

[9] Jiang SM, Cieslewicz MJ, Kasper DL, Wessels MR (2005). Regulation of virulence by a two-component system in Group B Streptococcus.. J Bacteriol.

[10] Jiang SM, Ishmael N, Hotopp JD, Puliti M, Tissi L (2008). Variation in the Group B Streptococcus CsrRS regulon and effects on pathogenity.. J Bacteriol.

[11] Poyart C, Lamy MC, Boumaila C, Fiedler F, Trieu-Cuot P (2001). Regulation of D-alanyl-lipoteichoic acid biosynthesis in *Streptococcus agalactiae* involves a novel two-component regulatory system.. J Bacteriol.

[12] Spellerberg B, Pozdzinski E, Martin S, Weber-Heynemann J, Lütticken R (2002). *rgf* encodes a novel two-component signal transduction system of *Streptococcus agalactiae*.. Infect Immun.

[13] Dmitriev A, Yang YN, Shen AD, Totolian A (2006). Adjacent location of *bac* gene and two-component regulatory system genes within the putative *Streptococcus agalactiae* pathogenicity island.. Folia Microbiol (Praha).

[14] Tettelin H, Masignani V, Cieslewicz MJ, Donati C, Medini D (2005). Genome analysis of multiple pathogenic isolates of *Streptococcus agalactiae*: implications for the microbial “pan-genome”.. Proc Natl Acad Sci U S A.

[15] Areschoug T, Stalhammar-Carlemalm M, Karlsson I, Lindahl G (2002). Streptococcal β protein has separate binding sites for human factor H and IgA-Fc.. J Biol Chem.

[16] Hoskins JA, Alborn W, Arnold J, Blaszczak L, Burgett S (2001). Genome of the bacterium *Streptococcus pneumoniae* strain R6.. J Bacteriol.

[17] Lanie JA, Ng WL, Kazmierczak KM, Andrzejewski TM, Davidsen TM (2007). Genome sequence of avery's virulent serotype 2 strain D39 of *Streptococcus pneumoniae* and comparison with that of unencapsulated laboratory strain R6.. J Bacteriol.

[18] Dopazo J, Mendoza A, Herrero J, Caldara F, Humbert Y (2001). Annotated draft genomic sequence from a *Streptococcus pneumoniae* type 19F clinical isolate.. Microb Drug Resist.

[19] Tettelin H, Nelson KE, Paulsen IT, Eisen JA, Read TD (2001). Complete genome sequence of a virulent isolate of *Streptococcus pneumoniae*.. Science.

[20] Jedrzejas MJ (2001). Pneumococcal virulence factors: structure and function.. Microbiol Mol Biol Rev.

[21] Murdoch C, Read RC, Zhang Q, Finn A (2002). Choline-binding protein A of *Streptococcus pneumoniae* elicits chemokine production and expression of intercellular adhesion molecule 1 (CD54) by human alveolar epithelial cells.. J Infect Dis.

[22] Standish AJ, Stroeher UH, Paton JC (2005). The two-component signal transduction system RR06/HK06 regulates expression of *cbpA* in *Streptococcus pneumoniae*.. Proc Natl Acad Sci U S A.

[23] Ma Z, Zhang JR (2007). RR06 Activates Transcription of spr1996 and *cbpA* in *Streptococcus pneumoniae*.. J Bacteriol.

[24] Standish AJ, Stroeher UH, Paton JC (2007). The pneumococcal two-component signal transduction system RR/HK06 regulates CbpA and PspA by two distinct mechanisms.. J Bacteriol.

[25] ManiatisT, Fritsch EE, Sambrook J (1982). Molecular cloning. A laboratory manual..

[26] Johnson DR, Kaplan EL, Sramek J, Bicova R, Havlicek J (1996). Laboratory diagnosis of group A streptococcal infections..

[27] Grubb AO, Grubb RE, Christensen PB, Shalen C (1982). Isolation and some properties of an IgG binding protein from group A streptococci type 15.. Int Arch Allergy Appl Immunol.

[28] Laemmli UK (1970). Cleavage of structural proteins during the assembly of the head of bacteriophage T4.. Nature.

[29] Towbin H, Staehelin T, Gordon J (1992). Electrophoretic transfer of proteins from polyacrylamide gels to nitrocellulose sheets: procedure and some applications. 1979.. Biotechnology.

[30] Panda B, Iruretagoyena I, Stiller R, Panda A (2009). Antibiotic resistance and penicillin tolerance in ano-vaginal group B streptococci.. J Matern Fetal Neonatal Med.

[31] Dogan B, Schukken YH, Santisteban C, Boor KJ (2005). Distribution of serotypes and antimicrobial resistance genes among *Streptococcus agalactiae* isolates from bovine and human hosts.. J Clin Microbiol.

[32] Kong F, Gidding HF, Berner R, Gilbert GL (2006). Streptococcus agalactiae Cbeta protein gene (bac) sequence types, based on the repeated region of the cell-wall domain: relationship to virulence and a proposed standardized nomenclature.. J Med Microbiol.

[33] Framson PE, Nittayajarn A, Merry J (1997). New genetic techniques for group B streptococci: high-efficiency transformation, maintenance of temperature-sensitive pWV01 plasmids, and mutagenesis with Tn917.. Appl Environ Microbiol.

[34] Dmitriev A, Tkáciková L, Suvorov A, Kantíková M, Mikula I (1999). Comparative genetic study of group B streptococcal strains of human and bovine origin.. Folia Microbiol.

[35] Berner R, Ruess M, Bereswill S, Brandis M (2002). Polymorphisms in the cell wall-spanning domain of the C protein beta-antigen in clinical *Streptococcus agalactiae* isolates are caused by genetic instability of repeating DNA sequences.. Pediatr Res.

[36] Nagano N, Nagano Y, Taguchi F (2002). High expression of a C protein beta antigen gene among invasive strains from certain clonally related groups of type Ia and Ib group B streptococci.. Infect Immun.

[37] Dmitriev A, Hu YY, Shen AD, Suvorov A, Yang YH (2002). Chromosomal analysis of group B streptococcal clinical strains; *bac* gene-positive strains are genetically homogenous.. FEMS Microbiol Lett.

[38] Leont'eva GF, Suvorov AN, Meringova LF, Grabovskaia KB, Ustinovich IA (2005). Experimental evaluation of recombinant polypeptides as Streptococcus Group B vaccine.. Zh Mikrobiol Epidemiol Immunobiol.

[39] Nagano N, Nagano Y, Nakano R, Okamoto R, Inoue M (2006). Genetic diversity of the C protein beta-antigen gene and its upstream regions within clonally related groups of type Ia and Ib group B streptococci.. Microbiology.

[40] Brady LJ, Boyle P (1989). Identification of non-immunoglobulin A-Fc-binding forms and low-molecular-weight secreted forms of the group B streptococcal beta antigen.. Infect Immun.

[41] Hynes W, Johnson C, Stokes M (2009). A single nucleotide mutation results in loss of enzymatic activity in the hyaluronate lyase gene of *Streptococcus pyogenes*.. Microb Pathog.

[42] Graham MR, Smoot LM, Migliaccio CA, Virtaneva K, Sturdevant DE (2002). Virulence control in group A Streptococcus by a two-component gene regulatory system: global expression profiling and in vivo infection modeling.. Proc Natl Acad Sci U S A.

[43] Rogasch K, Rühmling V, Pané-Farré J, Höper D, Weinberg C (2006). Influence of the two-component system SaeRS on global gene expression in two different *Staphylococcus aureus* strains.. J Bacteriol.

[44] Jerlstrom PG, Chhatwal GS, Timmis KN (1991). The IgA-binding beta antigen of the c protein complex of Group B streptococci: sequence determination of its gene and detection of two binding regions.. Mol Microbiol.

